# Web-Based Multidomain Lifestyle Programs for Brain Health: Comprehensive Overview and Meta-Analysis

**DOI:** 10.2196/12104

**Published:** 2019-04-09

**Authors:** Linda MP Wesselman, Astrid M Hooghiemstra, Linda J Schoonmade, Marjolein CJ de Wit, Wiesje M van der Flier, Sietske AM Sikkes

**Affiliations:** 1 Alzheimer Center Amsterdam Department of Neurology, Amsterdam Neuroscience Vrije Universiteit Amsterdam, Amsterdam UMC Amsterdam Netherlands; 2 Department of Medical Humanities Amsterdam Public Health Research Institute Vrije Universiteit Amsterdam, Amsterdam UMC Amsterdam Netherlands; 3 University Library Vrije Universiteit Amsterdam Amsterdam Netherlands; 4 Department of Epidemiology and Biostatistics Amsterdam Neuroscience Vrije Universiteit Amsterdam, Amsterdam UMC Amsterdam Netherlands; 5 Department of Neurology Massachusetts General Hospital Harvard Medical School Boston, MA United States

**Keywords:** meta-analysis, telemedicine, internet, lifestyle, healthy aging, cognition, dementia, health promotion, primary prevention

## Abstract

**Background:**

The number of people living with dementia is increasing worldwide, mainly because of aging of the population. To date, there is no pharmaceutical intervention to delay or treat cognitive decline or dementia. As an estimated one-third of dementia cases might be attributable to modifiable lifestyle factors (such as cognitive and physical activity), multidomain lifestyle interventions are a promising way to maintain or improve brain health. Offering programs online would enable large-scale implementation. An overview of multidomain Web-based lifestyle programs for brain health would facilitate comparison and improvement of such programs to develop effective and sustainable interventions.

**Objective:**

This study aimed to (1) provide a comprehensive overview of Web-based multidomain lifestyle programs aimed at optimizing brain health in healthy adult populations and (2) describe the programs and targeted lifestyle factors, availability, and evaluation of adherence and user experience. In addition, a meta-analysis was performed to evaluate the effectiveness of these programs.

**Methods:**

Electronic databases (PubMed, EMBASE, and PsycINFO) were searched for Web-based lifestyle programs that were included when the program (1) aimed to optimize brain health, (2) focused on multiple lifestyle factors, (3) was completely Web-based (website, Web application or mobile app), (4) consisted of multiple sessions, and (5) focused on a healthy adult population. Program characteristics (target population, duration, frequency, tailoring, platform, and availability) and results of program evaluations (effectiveness, user evaluations, and adherence) were extracted and compared. Studies using a controlled design were included in a random-effects meta-analysis on the effectiveness on brain health outcomes. Study quality was assessed using the physiotherapy evidence database (PEDro) scale.

**Results:**

The electronic searches yielded 44 documents describing 14 Web-based lifestyle programs; physical and cognitive activities were targeted in all programs. Four programs (4/14, 29%) were publicly available and free of charge, whereas others were restricted to research settings (5/14, 36%), available after payment (1/14, 7%), or not available at all (2/14, 14%). User evaluations were reported for 8 (57%) of the 14 programs. Reported dropout of the intervention groups ranged from 2% to 52%. Overall, 3 studies evaluated the effectiveness of a program using a controlled design and were included in the meta-analysis (moderate-to-high quality). Pooled results showed a significant small-to-medium effect of the Web-based multidomain lifestyle interventions on outcome measures for brain health (global cognition score, subjective cognitive score, and lifestyle risk score; standard mean difference=0.45; 95% CI 0.12-0.78), with a high degree heterogeneity across studies (I^2^=75%; *P*=.02).

**Conclusions:**

In total, 14 Web-based multidomain lifestyle programs aimed at optimizing brain health were found. The programs showed heterogeneity in both characteristics and effectiveness evaluation. Despite this heterogeneity, this meta-analysis suggests that Web-based lifestyle programs can positively influence brain health outcomes and have the potential to contribute to the prevention of dementia.

## Introduction

### Background

Dementia is characterized by a gradual decline of cognitive functioning and impairment in daily functioning [1]. Several neurodegenerative diseases can cause dementia, of which Alzheimer disease (AD) is the most common [2,3]. In 2015, 46 million people were living with dementia worldwide, and this number is estimated to increase to 131.5 million by 2050 [4] because of aging of the society [5]. Dementia causes a large burden on society and health care, with a worldwide estimated cost of $818 billion in 2015 [4,5].

To date, there is no pharmaceutical intervention to delay or treat cognitive decline or dementia. It is estimated that 30% of dementia cases are attributable to modifiable risk factors, including lifestyle factors such as physical activity, social activity, mood, and smoking [6-8]. Therefore, lifestyle interventions aimed at improving brain health and cognitive functioning before the onset of dementia (ie, in healthy persons) might delay or even prevent the occurrence of cognitive decline or dementia [6-9].

Studies on lifestyle interventions and cognition are mainly observational, with relatively limited evidence from large randomized trials [9]. However, evidence indicates that simultaneously targeting multiple lifestyle factors increases efficacy compared with programs focusing on a single lifestyle factor [10-12]. Furthermore, 2 nonsystematic reviews [10,13] and a systematic review with a meta-analysis [14] studied the effect of multidomain face-to-face interventions on cognitive outcomes. These latter studies reported that multidomain lifestyle interventions exerted overall positive effects on cognitive outcomes in healthy older people and in individuals with subjective cognitive decline. The Finnish Geriatric Intervention Study to Prevent Cognitive Impairment and Disability (FINGER) trial, a 2-year face-to-face multidomain intervention study (ie, diet, exercise, cognitive training, and vascular risk monitoring), demonstrated maintained global cognitive functioning in a group of participants at risk for cognitive decline, compared with a control group receiving general health advice [15].

Despite their potential, practical barriers of these face-to-face programs include the requirement of training appropriate staff and resources such as location and personnel to provide the intervention. An attractive alternative is to offer lifestyle programs online, making use of the rapidly developing landscape of electronic health (eHealth) [16]. This strategy is appealing for several reasons. First, in most countries, the majority of people have access to the internet [17], allowing them to reach larger audiences than face-to-face interventions. Second, Web-based programs have the potential to be cost-effective in the long run, with relatively low additional costs per person [18]. Third, Web-based programs might be more easily accessible at any convenient time, fitting them more easily into daily schedules and thereby increasing adherence. Finally, Web-based programs generate and collect data that can be used to automatically tailor content, for example, based on changes in lifestyle, as assessed by questionnaires. Criteria could then be set beforehand and tracked by the system. Web-based programs are easier to tailor than face-to-face programs, which need human involvement to adapt or to choose a protocol. Tailored programs improve the personal relevance of the program itself and, thus, may increase both adherence and effectiveness [19,20].

A comprehensive overview and meta-analysis of Web-based lifestyle programs for brain health is currently lacking but is required to explore the potential of these programs to benefit brain health. An overview of the programs and their evaluation might contribute to the development and implementation of more successful programs and, thereby, enable more effective and sustainable Web-based interventions.

### Objectives

Therefore, this study provides an overview of Web-based multidomain lifestyle programs that aim to optimize brain health and focuses on program characteristics, current availability, evaluation of adherence, and user evaluations. In addition, a meta-analysis is performed on the effectiveness of Web-based programs on outcome measures for brain health.

## Methods

### Search Strategy

A systematic literature search based on the PRISMA statement was performed in collaboration with a medical librarian (LS). The databases of PubMed, EMBASE, and PsycINFO (via Ebsco) were examined from inception to June 5, 2018 (see Multimedia Appendix 1 for the full search strategy). Search terms included indexed terms from the thesaurus in PsycINFO, MeSH in PubMed, and EMtree in EMBASE as well as free-text terms. The references of the identified articles were searched for additional relevant publications. As the primary goal was to provide an overview of existing Web-based multidomain lifestyle programs, programs were included irrespective of the stage of their development or evaluation. Original research articles were included as well as conference abstracts, reviews, and doctoral theses mentioning (the development of) a program.

In addition, included were documents that described a program that (1) aimed to optimize brain health or cognitive functioning, as appeared from the aim of the study, program descriptions, or choice of outcome measures; (2) focused on multiple lifestyle factors; (3) was completely Web-based (website, Web application or app) and self-administered with no need for a visit to or from a health care professional; (4) consisted of multiple sessions (ie, not a single exercise); and (5) focused on a healthy adult population. Documents not available in the English language were excluded.

Furthermore, 2 independent researchers (LW and MW) screened the documents and used Covidence [21] to record the process. First, all documents were assessed for relevance based on the title and abstract. Selected documents were retrieved in full text and checked for eligibility criteria. Differences in judgment were resolved through a consensus procedure including an independent third reviewer (AH). Project websites mentioned in articles were searched for additional publications. In addition, the literature databases and Google were searched to identify additional publications on the included programs, using the name of the program and the authors. To assess availability of the identified programs, Google, Google Play Store, and Apple store were searched. If necessary, the authors were contacted for additional information, for example, regarding the availability of the program.

### Data Extraction

The electronic searches yielded 11,972 documents: 3571 in PubMed, 5106 in EMBASE, and 3295 in PsycINFO. After removing duplicates, 7537 titles remained and were screened based on title and abstract; of these, 7461 were excluded. The majority of the excluded documents did not describe an intervention or a Web-based program that could be self-administered without the need for a health care professional. Of the remaining 76 studies, the full-text documents were scrutinized, applying the inclusion and exclusion criteria. The final selection for this review comprised 44 documents describing 14 Web-based lifestyle programs (see Multimedia Appendix 2 for the systematic search flowchart; see Table 1 for retrieved documents and the programs they describe).

A total of 24 (24/44, 55%) journal articles, 19 (19/44, 43%) conference abstracts, and 1 (1/44, 2%) doctoral thesis were retrieved. Half of the journal articles (12/24; 50%; denoted by footnote a in Table 1) were original research articles investigating 10 (71%) of the 14 programs. For 4 of the programs (4/14, 29%), no original research article was found. Data were extracted based on program characteristics, target population, duration (length of intervention), frequency of the program (eg, number of sessions and number of modules), tailoring of content, lifestyle factors, platform of the program (website or app), current availability, and evaluation of the program.

### Evaluation of Web-Based Multidomain Lifestyle Programs

#### Effectiveness

Results of studies that used a controlled design were pooled to perform a meta-analysis on the effectiveness of Web-based multidomain lifestyle programs on outcome measures relevant for brain health or cognitive functioning. This resulted in a meta-analysis including 3 studies entitled *Body Brain Life* [23], *Keep your brain fit* [53], and *Long Lasting Memories* [58]. For these studies, 2 raters (LW and AH) assessed the study quality and risk of bias using the physiotherapy evidence database (PEDro) scale [65-67]. The PEDro scale evaluates the internal validity by assessing the eligibility criteria, allocation, blinding and reporting of outcome measures, between-group comparisons, and variability. For this review, we deemed the PEDro item *blinding of therapists* not to be applicable, as interventions were provided as self-administered on the internet and not by therapists.

#### User Evaluations

During the development and evaluation of Web-based interventions, the involvement of users for evaluation of *user experience* and *usability* is important; however, discussion continues as to what these terms exactly entail [68]. We based our definitions on the International Organization of Standardization (ISO) guidelines (ISO 9241-2010, ISO/ICE 25010) and selected parts of the guidelines to summarize the extracted information. First, *user experience* includes all the users’ emotions, beliefs, preferences, perceptions, physical and psychological responses, behaviors, and accomplishments that occur before, during, and after use of the Web-based program. Second, we defined *usefulness* as the users’ perception on whether the program served their needs and purpose and was helpful in any way. Finally, *usability* was defined as whether the program was convenient and easy to use, taking into account the technical aspects of the platform. For each study, we assessed whether our definitions of user experience, usefulness, or usability were described. Subsequently, we indicated whether methods used for this evaluation were clearly described, by defining whether it would be possible to replicate the design. *Qualitative debriefing* or *survey with open question* were deemed not specific enough, whereas specific questionnaire items that were used to collect information on user experience were deemed sufficient.

#### Adherence

Assessing adherence to the usage of an eHealth technology can be challenging, as also noted in a recent review [69]. However, because the majority of the programs in this review did not describe the intended use of the program, it was not possible to assess usage adherence (ie, not in terms of number of log-ins or time spent). Therefore, adherence was assessed as the percentage of participants completing the intervention and postintervention measurement or in relation to the number of participants that started the intervention.

**Table 1:** Characteristics of the 14 included Web-based lifestyle programs.

Number	Program (acronym and full name)	Target population	Program characteristics	Website or app	Availability
1	Body Brain Life: [22] + [23]^a^	Nonsymptomatic adults at risk for Alzheimer disease	Duration: 12 weeks; number of sessions: 12; frequency: 1/week; and tailored: yes, Lifestyle-Q^b^	Website	Available: yes; public: no
2	Brain Aging Monitor [24,25] + [26]^a^	Dutch workforce and general population	Duration: 52 weeks; number of sessions:—^c^; frequency:—; tailored: yes, Lifestyle-Q and goals set	Website	Available: no
3	Brainy app [27,28] + [29]^a^	General population	Duration: 4 weeks; number of sessions: —; frequency: —; tailored: yes, Lifestyle-Q	iOS app and Android app	Available: yes; public: yes; Free of charge: yes Link
4	Brain-Heart Health Plus program [27,28] + [29]^a^	General population	Duration: 4 weeks; number of sessions:—; frequency:—; tailored: yes, Lifestyle-Q	Website	Available: yes; but redesigned format; public: yes; free of charge: yes Link
5	DoReMi: Decrease of cOgnitive decline, malnutRition and sedEntariness by elderly empowerment in lifestyle Management and social Inclusion [30,31]	General population, elderly	Duration: 13 weeks; number of sessions: 36; frequency: 3/week; tailored: yes, acquired data	Android app	Available: No
6	Gray Matters [32-34] + [35]^a^	General population	Duration: 26 weeks; number of sessions: —; frequency: —; tailored: yes; and Lifestyle-Q	iOS app and Android app	Available: yes; public: no
7	HATICE: Healthy Aging Through Internet Counselling in the Elderly [12,36-47] + [48,49]^a^	Elderly with cardiovascular risk factors, cardiovascular disease or diabetes mellitus	Duration: 78 weeks; number of sessions: —; frequency: 1/week; tailored: yes, goals and topic interest	Website	Available: yes; public: no
8	InMINDD: INnovative, Midlife INtervention for Dementia Deterrence [50,51]	Individuals with 1 modifiable risk factor based on Lifestyle for Brain Health (LIBRA) score [52]	Duration: 26 weeks; number of sessions: —; frequency: —; tailored: yes, demographic, health behavior, and clinical information	Website	Available: yes; public: no
9	Keep your brain fit [53]^a^	General population	Duration: 4 weeks; number of sessions: —; frequency: —; tailored: yes, Lifestyle and cognition monitor’	Website	Available: yes; public: yes; free of charge: yes Link
10	LEAP: Lifestyle Enrichment for Alzheimer Prevention [54]^a^	Individuals with upcoming retirement	Duration: 8 weeks; number of sessions: —; frequency: —; tailored: yes, Lifestyle-Q and Goals	Website	Available: yes; public: yes; free of charge: yes Link
11	Long Lasting Memories [13,55-57] + [58,59]^a^	Older adults with or without neurocognitive disorders	Duration: 10 weeks; number of sessions: 24; frequency: 4/week; tailored: yes, patient group	Website + Android or iOS app for cognitive component	Available: yes; public: no; free of charge: no; physical component: €100/6 months, cognitive component: €100/6 months Link
12	Maintain your brain [60,61]	Adults with 2 risk factors for dementia	Duration: 208 weeks (4 years); number of sessions: (4x10-week modules in the first year); frequency: —; tailored: yes, current lifestyle	—	Available: Yes; public: no
13	Smart Aging [62]	General population, seniors	Duration: 16 weeks; number of sessions: —; frequency: 7/week; and tailored: —	—	—
14	Vital Aging Program (e-learning version) [63,64]^a^	Population based (students from university programs for older adults)	Duration: 13 weeks; number of sessions: —; frequency: 1/week; and tailored: —	Website	—

^a^Original research articles.

^b^Lifestyle-Q: Lifestyle Questionnaire.

^c^Information that was not specified.

### Statistical Analysis and Meta-Analysis

IBM SPSS Statistics (version 22) [70] was used to calculate descriptive statistics to summarize program characteristics. For the studies that assessed effectiveness, postintervention means, SDs, and group size were pooled. We used the results that were reported for the primary outcome measure that assessed brain health or cognitive functioning (ie, objective and subjective measures). Results were inverted if necessary, making higher scores represent better scores. Review Manager (version 5.3, The Cochrane Collaboration, Copenhagen, Denmark) was used to perform a random-effects meta-analysis. The overall effect of lifestyle interventions on brain health outcomes was estimated, and the results were presented as a forest plot. The Cochrane Χ^2^ was used to test for heterogeneity across the included articles, with a *P*<.05 indicative of heterogeneity. The I^2^ (100 x (Χ^2^ - df/Χ^2^) [71] was used to measure the degree of heterogeneity (25%, low; 50%, moderate; and 75%, high [72]).

### Preferred Reporting Items for Systematic Reviews and Meta-Analysis Statement

As the scientific output in this field is somewhat limited, not all the items of the Preferred Reporting Items for Systematic Reviews and Meta-Analysis (PRISMA) statement were deemed applicable. Information that contributed to the aim of this review is reported and includes the characteristics, availability, and evaluation.

## Results

### Characteristics of the Web-Based Multidomain Lifestyle Programs

Table 1 provides an overview of the 14 included Web-based lifestyle programs (with full names and their characteristics). Overall, 8 programs (8/14, 57%) were developed for a general adult population (*Brain Aging Monitor* [24], *Brainy app* [29], *Brain-Heart Health Plus Program* [29], *Decrease of cOgnitive decline, malnutRition and sedEntariness by elderly empowerment in lifestyle Management and social Inclusion (DoReMi)* [30], *Gray Matters* [35], *Keep your brain fit* [53], *Smart Aging* [62], and *Vital Aging program* [63]). In addition, 6 programs (6/14, 43%) described a more specific target population: *Body Brain Life* [22] and *Maintain your Brain* [60] focused on nonsymptomatic adults at risk of AD, *Healthy Aging Through Internet Counselling in the Elderly (HATICE)* [36] targets elderly with cardiovascular risk factors and cardiovascular disease, and *INnovative, Midlife INtervention for Dementia Deterrence (InMINDD)* [50] targets individuals with 1 modifiable risk factor. The *Lifestyle Enrichment for Alzheimer Prevention (LEAP)* [54] program was developed for individuals with upcoming or recent retirement and *Long Lasting Memories* [58] for individuals with or without a cognitive impairment.

The programs were offered on a website (8/14, 57% [22,24,29,36,50,53,54,63]), a mobile app (3/14; 21% [29,30,35]), or both (1/14, 7% [55]), whereas the platform was not specified for the remaining 2 [60,62]. Only 4 (4/14, 29%) programs were available publicly and free of charge [29,53,54]. Overall, 5 programs (5/14, 36%) were available within a research setting [22,35,36,50,60] and 1 (1/14, 7%) after payment [58]. In addition, 2 programs (2/14, 14%) were not available online at all [25,30], and for 2 programs (2/14, 14%), it was not clear whether they were still available [62,63]. Mean intervention duration was 33.9 (SD 54.1; range 4-204) weeks. Furthermore, 3 programs [22,30,55] specified the total number of sessions (mean 24, [SD 12]; range 12-36 sessions), and 6 programs [22,30,36,55,62,63] indicated a frequency per week (mean 2.8 [SD 2.4]; range 1-7 sessions/week). For the remaining programs, the number of sessions and the frequency per week were flexible.

Most programs tailored the content of the program to previously acquired information. Content was mainly tailored based on current lifestyle (eg, assessed with a questionnaire on lifestyle and risk factors; 9/14, 64% [22,24,29,35,36,50,53,60]). Furthermore, 3 programs (3/14, 21%; [25,36,54]) additionally tailored content based on goals that were set within the program, and 2 programs (2/14, 14%; [62,63]) did not specify information on tailoring. The number of lifestyle factors targeted in the programs ranged from 2 [58] to 9 [60] (Table 2). In short, all interventions targeted a physical and a cognitive component. Furthermore, most programs included a nutritional component (13/14 programs, 93%; all except Long Lasting Memories [58]) as well as a social component (10/14 programs, 71%; [22,29,30,35,50,53,54,62,63]). Half of the programs included smoking cessation (7/14, 50%; [24,29,36,50,60,62]), and other factors included alcohol intake, vascular risk factors, stress management, sleep, and mood.

**Table 2:** Lifestyle factors targeted in the included programs. Per row, the factors per program are presented. Per column, the programs including this specific lifestyle factors are presented.

Program	Physical	Cognitive	Nutrition	Social	Smoke	Vascular^a^	Alcohol	Stress	Sleep	Mood	Other^b^
Body Brain Life	x^c^	x	x	x	—	—	—	—	—	—	x
Brain Aging Monitor	x	x	x	—	x	—	x	x	x	—	—
Brainy app	x	x	x	x	x	x	x	—	—	—	—
Brain Heart Health Plus Program	x	x	x	x	x	x	x	—	—	—	—
Decrease of cOgnitive decline, malnutRition and sedEntariness by elderly empowerment in lifestyle Management and social Inclusion	x	x	x	x	—	—	—	—	—	—	—
Gray Matters	x	x	x	x	—	—	—	x	x	—	—
Healthy Aging Through Internet Counseling in the Elderly	x	x	x	—	x	x	—	—	—	—	—
INnovative, Midlife INtervention for Dementia Deterrence	x	x	x	x	x	x	x	—	—	x	—
Keep your brain fit	x	x	x	x	—	—	—	x	x	x	—
Lifestyle Enrichment for Alzheimer Prevention	x	x	x	x	—	—	—	—	—	—	x
Long Lasting Memories	x	x	—	—	—	—	—	—	—	—	—
Maintain your brain	x	x	x	—	x	x	x	x	x	x	—
Smart Aging	x	x	x	x	x	x	—	—	—	—	—
Vital Aging Program	x	x	x	x	—	—	—	—	—	x	x
Total number of programs targeting the lifestyle factor	14	14	13	10	7	6	5	4	4	4	3

^a^Vascular: this category summarized vascular and physical variables often used in vascular risk management, for example, blood pressure and weight.

^b^Other factors included were for Body Brain Life: dementia literacy, dementia risk factors, and health self-management; for Lifestyle Enrichment for Alzheimer Prevention: time management, and for Vital Aging Program: body caring.

^c^Per column it is indicated whether a program does (x) or does not (—) include the specific lifestyle factors.

### Evaluation of Web-Based Multidomain Lifestyle Programs

#### Effectiveness, Study Quality, and Meta-Analysis

Effectiveness was measured on a range of brain health outcomes, such as an AD risk questionnaire (eg, lifestyle factors associated with the development of AD such as food intake or level of physical activity), cognitive test performance, and the subjective experience of cognitive problems. Table 3 presents an overview of the outcome measures.

For 4 programs (4/14, 29%), effectiveness was evaluated, of which 3 (3/14, 21%) used a controlled design. The latter 3 were included in the meta-analysis: *Body Brain Life* [23], *Keep your brain fit* [53], and *Long Lasting Memories* [58] (not included [26,59]). On the PEDro scale, the quality of the studies ranged from moderate (5/10 [58]) to high (8/10 [23]; Table 4). All 3 studies specified recruitment of participants and eligibility criteria. At baseline, the experimental groups were comparable with the control groups. In addition, all used an intention-to-treat analysis and reported between-group comparisons as well as point estimates with measures of variability. Points were lost on the PEDro items for randomization, blinding of subjects and assessors, and the cutoff of 85% of participants completing follow-up measurement.

**Table 3:** Outcome measures. The outcome measures per study are shown, as described in the retrieved documents. If authors delineated primary and secondary outcome measures, this is indicated in the column *Priority*. For primary outcome measures, the specific outcome measure is described.

Program	Priority	Outcome measure per domain	Specific outcome measure
Body Brain Life	Primary	Lifestyle-risk factors-Q^a^	ANU Alzheimer's Disease Risk Index (ANU-ADRI) [73]
Body Brain Life	Secondary	Cognition: objective; body measurements; other: dementia recognition, and dementia knowledge	—^b^
Brain Aging Monitor	Primary	Cognition: objective; Lifestyle-risk factors-Q; and Goal setting	BAM-COG games [74] Lifestyle score (including physical activity, nutrition, and sleep) [24]
Brain Aging Monitor	Secondary	Body measurements and other: change in self-efficacy and change in self-control	—
Brainy app + Brain-Heart Health Plus program (not prioritized)	—	Lifestyle-risk factors-Q and other: Dementia risk reduction knowledge and attitudes to changing behavior	Not specified; 10 factors (risk/protective for dementia); eg, motivation to reduce dementia risk
Decrease of cOgnitive decline, malnutRition and sedEntariness by elderly empowerment in lifestyle Management and social Inclusion (not prioritized)	—	Cognition: objective; Lifestyle: measurement and Body measurements	Montreal Cognitive Assessment (MoCA) [75], Token test [76]; Mini Nutritional Assessment (MNA) (version not specified) [77]; BMI^c^, waist-to-hip ratio, waist and arm circumference, and blood pressure related to the 6-min walk test
Gray matters	Primary	Body measurements and Lifestyle-risk factors-Q	BMI, blood pressure, blood biomarkers including physical and cognitive activity, nutrition (Diet History Questionnaire + DASH score), sleep, social engagement, and stress
Gray matters	Secondary	Lifestyle-risk factors-measurement; cognition: objective; other: mood and psychological stress; metacognition, intrinsic motivation; readiness to change, sleep quality; and social engagement, couple satisfaction	—
Healthy Aging Through Internet Counselling in the Elderly	Primary	Body measurements	Composite score (systolic. blood pressure, low-density lipoprotein, and BMI)
Healthy Aging Through Internet Counselling in the Elderly	Secondary	Body measurements; lifestyle-risk factors-Q; cognition: objective; clinical measurement; goal setting; other: mood, cost-effectiveness	—
INnovative, Midlife INtervention for Dementia Deterrence	Primary	Lifestyle-risk factors-Q	Lifestyle for Brain Health (LIBRA) score [52]
INnovative, Midlife INtervention for Dementia Deterrence	Secondary	Lifestyle-risk factors-Q	—
Keep your brain fit	Primary	Cognition: subjective	Subj cognitive functioning (MIA) [78], cognitive failure (CFQ; [79,80]), Self-evaluative questions [81]
Keep your brain fit	Secondary	Cognition: objective; other: Depression, anxiety, stress, Self-rated health, Feelings of loneliness, Difficulties in recovering from work, General health, and QoL^d^	—
Lifestyle Enrichment for Alzheimer Prevention	Primary	Lifestyle-risk factors: measurements	Dietary intake [82] and physical activity (accelerometry); weight, BMI, waist circumference, body fat mass, fat-free mass, today body water, body weight, and waist circumference; and cognition, physical capability, physiological, and psychosocial well-being
Lifestyle Enrichment for Alzheimer Prevention	Secondary	Body measurements; Battery of Healthy aging phenotype (HAP) measurements; and other	—
Long Lasting Memories (not prioritized)	—	Body measurements; cognition: objective; Lifestyle-risk factors-Q; and other: mood, Quality of Life, IADL	Physical fitness (composite score Senior fitness test); eg, Mini Mental State Examination, MoCA, Trail Making Test, California Verbal Learning Test; and Social Life Questionnaire
Maintain your brain	Primary	Cognition and clinical measurement	Unspecified and Dementia incidence
Maintain your brain	Secondary	Lifestyle-risk factors-Q; goal setting; clinical measurements; and medication	—
Smart Aging	—	Other: QoL	—
Vital Aging Program (e-learning)	—	Other: reported changes and intended future changes	—

^a^Q: Questionnaire.

^b^Information either not available (priority) or not specified (secondary outcome measures).

^c^BMI: body mass index.

^d^QoL: quality of life.

**Table 4:** Quality assessment of pooled studies. This table presents the quality and bias assessment of the studies that were pooled in the meta-analysis, based on the physiotherapy evidence database scale (PEDro). Blinding of therapist was deemed not applicable for all 3 studies, as the interventions were offered by automated systems. Concealed allocation was deemed not applicable for Long Lasting Memories, as participants were not randomized. Blinding of assessors was deemed for Keep your brain fit, as the assessment was conducted within the Web-based system.

Items	Body Brain Life [23]	Keep your brain fit [53]	Long Lasting Memories [58]
Eligibility criteria and source specified	+^a^	+	+
Random allocation	+	+	−^b^
Concealed allocation	+	−	N/A^c^
Baseline comparability	+	+	+
Blinding of subjects	−	−	−
Blinding of therapists	N/A	N/A	N/A
Blinding of assessors	+	N/A	−
More than 85% follow-up	−	−	−
Intention-to-treat analysis	+	+	+
Reporting of between-groups statistical comparisons	+	+	+
Reporting of point measures and measures of variability	+	+	+
Total PEDro score 0-10	8	6	5

^a^Criteria fulfilled.

^b^Criteria not fulfilled or it was unclear whether criteria were fulfilled.

^c^Criteria not applicable.

Directly after the intervention, participants of *Body Brain Life* [23] showed no significant reduction in risk for AD compared with the control group. However, at 26-week follow-up, the intervention group showed a significant reduction in risk compared with the control group (intervention group mean score pretest=−1.07 [SD 0.72], posttest=−3.63 [SD 0.77]; control group pretest=−1.38 [SD 0.70], posttest=−1.93 [SD 0.73]; beta: −.37, SE 0.16, *P*=.05). This result was mainly because of an increase in protective lifestyle behaviors and not because of a decrease in risk factors, such as smoking or high cholesterol. For *Keep your brain fit* [53], there was a significant effect of group on perceived change in memory functioning (intervention group mean score pretest=29.99 [SD 6.22], posttest=31.46 [SD 6.06]; control group pretest=28.97 [SD 7.50], posttest=30.17 [SD 7.05]; *P*=.007), showing a small effect (Cohen *d*=.20). Moreover, a significant effect of group was found on a cognitive failure questionnaire (intervention group mean score pretest=66.15 [SD 11.36]; posttest=65.85 [SD 10.30]; control group pretest=63.76 [SD 11.98], posttest=65.11 [SD 12.25]; *P*=.03); however, this could be explained by baseline group differences. There was no significant effect of group on perceived memory capacity. Compared with an active control group, the participants of *Long Lasting Memories* [58] showed a significant improvement in global cognition (*t*_219_=3.2; *P*=.002), with a medium effect (Cohen *d*=.31). The cognitive status of the participants of *Long Lasting Memories* ranged from healthy to dementia, and exploratory analysis in the effectiveness study [58] indicated that the effect did not differ for the different diagnostic groups; therefore, this latter study is included in the meta-analysis, despite the inclusion of cognitively impaired participants.

**Figure 1:**
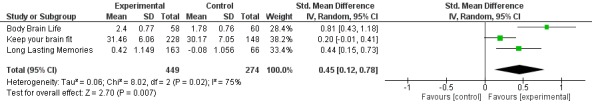
Forest plot meta-analysis. This figure presents the results of the random-effect meta-analysis that included data from 3 effectiveness studies using a controlled design. Outcome measures were measures for brain health (Body Brain Life: ANU Alzheimer's Disease Risk Index and lifestyle risk score; Keep your brain fit: Meta Memory in Adulthood scale and subjective cognitive functioning, and Long Lasting Memories: Mini Mental State Examination and global cognition score). Duration of the interventions: Body Brain Life, 12 weeks; Keep your brain fit, 4 weeks; Long Lasting Memories, variable, with an average of 6 weeks.

Results of these 3 studies were pooled in a meta-analysis, combining a lifestyle risk score (ANU Alzheimer's Disease Risk Index Questionnaire [73] for *Body Brain* Life [23]), a subjective cognitive measure (Meta memory in Adulthood Questionnaire [78] for *Keep your brain fit* [53]), and a global cognitive measure (Mini Mental State Examination [83] for *Long Lasting Memories* [58]). Pooled results (see Figure 1) showed a significant overall small-to-medium effect of the Web-based multidomain lifestyle interventions on outcome measures for brain health or cognitive functioning in the intervention group compared with the control group (standardized mean difference [SMD]=0.45; 95% CI 0.12-0.78). The degree of heterogeneity across studies was high (I^2^=75%; *P*=.02).

#### User Evaluations

For 8 programs (8/14, 57%), 10 studies [29,32,35,36, 48,49,53,54,62,63] reported user evaluations: all 10 reported results on usability and 7 (7/14, 50%) also reported results on usefulness and usability (for evaluations see Table 5). The methods to evaluate user experiences, usefulness, and usability were qualitative debriefing, a survey, Likert scales ranging from 0 to 10 for specific topics (eg, satisfaction and simplicity), and the percentage of participants that reported the program to be usable and user-friendly. User evaluations offered input regarding content and technical features to improve the Web-based multidomain lifestyle programs. Overall, the programs were evaluated as usable. In general, the reported barriers were mainly technical, such as password setting and navigation method. In addition, 2 studies evaluating the user-based concepts of 3 programs described their methods clearly and in a reproducible way [29,54]. In 1 study, the *Brain Heart Health Plus Program* and the *Brainy app* were compared directly [35]. Most participants evaluated both programs as overall positive and reported that the information was interesting, easy to understand, easy to navigate, and insightful. Users of the *Brain Heart Health Plus Program* website were more positive than users of the app, which the authors attributed to the difference in platform and the lack of instructions on how to use the app.

In their evaluation of *LEAP* [54], participants rated the modules on physical activity, social activity, and eating well as the highest; design, navigation, and technical issues were problematic for a few users.

The other 5 programs described results of user evaluations using a variety of methods: a few representative findings are presented here. Participants of Gray Matters [32,35] used the app regularly, with on average 3 app launches per week, and ranked 6 behavioral domains in order of importance: physical activity, cognitive stimulation, healthy food choices, stress management, sleep quality, and social engagement. Participants of *HATICE* [36,49] provided input on the password difficulty and deemed an instruction video necessary. Interactive features and healthy lifestyle content were valued, and participants liked to print the content. Using interviews, user-friendliness, usefulness, and perceived benefit were identified as important factors for initial use. Expectation of, and experience with lifestyle changes, and incorporation into daily routine were deemed important for sustained platform use [48]. Participants of *Keep your brain fit* [53] evaluated the program with a mean score of 7.3 (SD 1.09) out of 10 (n=228). Most recommendations for improving the intervention were technical (eg, more time to complete the intervention or more reminders) or content related (better explanation of the concepts). For the *Vital Aging Program*, most participants reported that the course was interesting, expectations were sufficiently met, and the content was helpful to improve their daily living (95.8%, 94%, and 96%, respectively) [63].

#### Adherence and Dropout

Overall, 6 studies included data on adherence to the intervention period [23,26,29,53,54,58]. In total, 1455 (1455/3598, 40.44%) participants dropped out of the active programs before completing the intervention period and postintervention measurement (mean 243 [SD 473.9]; range 1-1205 participants). The high dropout rate was mainly because of the study on the *Brain Aging Monitor* [26] (1205/2305, 52,27%). Excluding this study, a total of 250 (250/1293, 19.33%) participants dropped out (mean 50 [SD 53.4]; range 1-128 participants). The dropout rates of the intervention group of the other studies were 2% (1/58; Body Brain Life [23]), 10.8% (45/415; Brainy app and Brain Heart Health Plus program combined [29]), 36.0% (128/356; Keep your brain fit [53]), 4% (2/50; LEAP [54]), and 31.2% (74/237; Long Lasting Memories [58]). Reported reasons for dropping out were time constraints, dissatisfaction with the content, family issues, and physical illness. Dropouts were younger, had a higher education level, were more likely to work full time, and had a job that required mental and physical activity [29,53]. The other programs found no differences between the completers and noncompleters.

**Table 5:** User evaluations. This table shows whether the concepts of user experience, usefulness, and usability were evaluated and whether the evaluation methods were clearly described. For definitions, see Methods section User Evaluations.

Program	User experience	Usefulness	Usability
Body Brain Life	−^a^	−	−
Brain Aging Monitor	−	−	−
Brainy app [29]	+^b^	+	+
Brain Heart Health Plus Program [29]	+	+	+
Decrease of cOgnitive decline, malnutRition and sedEntariness by elderly empowerment in lifestyle Management and social Inclusion	−	−	−
Gray Matters [32,35]	−	~^c^	~
Healthy Aging Through Internet Counselling in the Elderly [36,48,49]	+	+	~
INnovative, Midlife INtervention for Dementia Deterrence	−	−	−
Keep your brain fit [53]	~	−	~
Lifestyle Enrichment for Alzheimer Prevention [54]	+	+	+
Long Lasting Memories	−	−	−
Maintain your brain	−	−	−
Smart Aging [62]	~	~	~
Vital Aging Program [63]	~	~	~

^a^User-based concepts were not evaluated or it was unclear whether the concepts were evaluated.

^b^Results were described and methods were specified.

^c^Results were described but methods were unspecified.

## Discussion

### Principal Findings

In this systematic review of Web-based multidomain lifestyle programs, 14 programs that aimed to optimize brain health were found. Comparison of these programs showed strong heterogeneity between program characteristics, targeted lifestyle factors, and program duration. In addition, detailed information on user evaluation methods and results was often lacking. Pooling of 3 studies that evaluated the effect of a Web-based program [23,53,58] showed a small-to-medium beneficial effect of Web-based multidomain lifestyle interventions on brain health outcomes.

Our main finding that Web-based multidomain lifestyle interventions have a beneficial effect on brain health is in line with previous results from face-to-face lifestyle programs, both reporting modest effect sizes [84,85]. This indicates that Web-based programs have the potential to yield health benefits comparable with those of face-to-face interventions, although no head-to-head comparisons have been made.

For 3 programs, effectiveness was evaluated using a controlled design [23,53,58]. In this meta-analysis, heterogeneity between the studies was high, with notable differences in outcome measures. We pooled data from a global cognitive score, a subjective cognitive score, and a risk-score questionnaire. The study on *Long Lasting Memories* [58] included a group of individuals with heterogeneous cognitive status, ranging from healthy to dementia. Exploratory subgroup analyses showed that the effect of the total intervention group might be conservative and an underestimation for healthy individuals. Although our meta-analysis included individuals over the whole cognitive spectrum, it is noteworthy that the overall intervention effect might be larger in a group of solely healthy individuals.

All described programs stimulated both physical and cognitive activities, that is, 2 lifestyle factors that have been extensively investigated in both healthy and cognitively impaired individuals [86-88]. The content of the Web-based lifestyle programs could be extended by including other lifestyle factors such as smoking, mood, and social activity. Although their influence on brain health may be less well understood, literature suggests that they could be part of a lifestyle that is beneficial for brain health and cognitive functioning [6].

With regard to the targeted lifestyle factors in the meta-analysis, 2 to 7 lifestyle factors (including physical and cognitive activity) were included in their programs. The program with the largest effect size (Body Brain Life [23]; SMD=0.81, 95% CI 0.43-1.18) targeted 4 lifestyle factors, whereas the program with the smallest effect size (Keep your brain fit [53]; SMD=0.20 95% CI −0.01 to 0.41) targeted 7 lifestyle factors. However, the duration of Body Brain Life was longer than Keep your brain fit (12 weeks and 4 weeks, respectively). Long Lasting Memories ([58]; SMD=0.44 95% CI 0.15-0.73) targeted 2 lifestyle factors, and, with a 10-week duration, it was in between the 2 latter programs. Due to the heterogeneity, no conclusions can be drawn about the specific successful factors of these programs, other than that at least a physical and cognitive activity were included in all programs and, therefore, seem beneficial for brain health. Better and head-to-head evaluations are necessary to compare the number of lifestyle factors, intervention duration, and potential synergistic effects.

Overall, the results of this study are promising, as small-to-medium effect sizes may translate to large public health gains when implemented on a large scale [19,20]. In particular, in the context of a global health care burden, given that a 1-year delay of onset of AD may translate to the prevention of 9 million cases worldwide in 2050, large-scale prevention with a small effect may be very cost effective [89].

Most of the included programs were not currently available for the general public. The majority were only available in a research setting, and 2 programs were not available online at all [25,30]. A sustainable implementation proves a key challenge in the eHealth field [90]. We need to create a bridge between the innovation in health care and the users in the general population. Proper education of health care professionals could increase the perceived usefulness and recommendation of eHealth innovations and, thereby, increase overall implementation success. Fitting the intervention to the needs of the user and making the intervention accessible could improve adherence rates and contribute to this sustainability. Therefore, it is recommended to involve users during every step of the developmental process [90,91]. Feedback from users helps to elucidate what a user experiences, which elements are appreciated, and/or to impose a boundary to participate. As concepts in the field of user evaluations are still evolving and used interchangeably [68], clear descriptions of methods and results could contribute to the comparability of the findings. In this review, half of the studies included user evaluations. However, the descriptions of user evaluations were often unclear, with an incomplete or lacking description of methods or lack of specification regarding which questions were asked to evaluate usability, usefulness, and user experience. In the studies that included user evaluations, facilitators for the use of the program were mentioned [29,32,35,48,62,63], such as a program being easy to understand, easy to navigate, and containing interesting content. Barriers that were most often mentioned were of a technical nature [36,49,53,54]. This highlights that the technical development of a program is just as important as the development of content. We recommend to develop the innovation step by step, together with both the technical team and the future users [90], to minimize technical barriers and create content that users deem interesting and useful. Technical features, such as a clear navigation or the possibility to receive reminders, are one of the aspects that influence adherence to a Web-based program.

Sustained adherence to Web-based programs is also an important issue in the eHealth field [92,93]. The adherence rates of the described studies were moderate to good and might be related to the tailoring of content [19,20]. Although most programs reported to be tailored, it was often unclear on what specific information tailoring was based and how the content was personalized based on this information. When comparing adherence rates between Web-based and face-to-face programs, we found comparable or higher adherence in face-to-face programs than in Web-based programs. For example, the face-to-face multidomain FINGER trial in elderly with cardiovascular risk factors for dementia [15] had high adherence rates (7% dropout at 12 months), whereas, for example, a face-to-face multidomain study in the frail elderly reported a 24% dropout mainly because of health problems [94]. Behavior change techniques and the role of communication with peers or a health coach might also influence the adherence rate. These aspects were not included in this review on self-administered programs and, because of the limited description of these aspects in the included documents, overall comparison of this matter with face-to-face programs was not possible.

Despite our extensive search, the small number of original research papers retrieved reflects the limited description of the included Web-based lifestyle programs for brain health and their evaluation. Descriptions might be improved by using a more rigorous design and report. To facilitate a standardized reporting, the CONSORT-EHEALTH (Consolidated Standards of Reporting Trials of Electronic and Mobile HEalth Applications and onLine TeleHealth) Group developed a checklist [95] that could be used during the development of eHealth interventions. This list includes recommendations on the design and, moreover, on the elements that should be included in the reporting of studies. Using this list may improve reporting and provide a basis for evaluation of the validity and applicability of eHealth trials, which might help the field to move forward.

### Strengths and Limitations

A limitation of this study is that the meta-analysis was performed on a small subset of studies. This limitation highlights the premature stage of Web-based lifestyle programs for brain health. Specific limitations of the meta-analysis include the heterogeneous study outcomes, differences in duration of the intervention, differences in the targeted lifestyle factors, and the heterogeneity in the sample of 1 study. However, based on the increasing use of eHealth and the need for dementia prevention strategies, more articles describing a Web-based multidomain lifestyle intervention and its evaluation are expected in the near future.

This overview of Web-based lifestyle programs for brain health was based on an elaborative search in 3 scientific databases, including journal papers as well as gray literature (eg, abstracts). The gray search was useful, as not all programs were described in the full research papers. We used a broad scope of search terms for the inclusion of studies, by widely applying inclusion criteria, for example, not specifying target populations. This broad scope contributes to the generalizability of the findings of this study. Screening of abstracts and full texts was performed by 2 independent raters and, if necessary, consensus meeting with a third rater took place, contributing to the reliability of our findings. Although the studies were highly heterogeneous, systematic elements were combined, resulting in a structured overview of programs and their evaluation. The subset included in the meta-analysis was small and heterogeneous, limiting generalization of the results. Nevertheless, the findings justify further exploration of the possibilities to implement Web-based lifestyle program to optimize brain health.

### Conclusions

In conclusion, we have provided a systematic overview and meta-analysis of studies on Web-based multidomain lifestyle programs to optimize brain health. Our findings suggest that these programs have a beneficial effect on brain health outcome measures. It would benefit the field if the program characteristics, methods, and results of evaluation of the programs were described in a more consistent way. This would facilitate comparison between programs and contribute to the development and implementation of effective and sustainable programs. Having shown their potential to optimize brain health in large groups of individuals, the implementation of Web-based lifestyle programs may well contribute to the prevention of dementia.

## Appendix

Multimedia Appendix 1 Search strategy.

Multimedia Appendix 2 Systematic search flow chart.

## Abbreviations

AD: Alzheimer disease
eHealth: electronic health
FINGER: Finnish Geriatric Intervention Study to Prevent Cognitive Impairment and Disability
HATICE: Healthy Aging Through Internet Counselling in the Elderly
ISO: International Organization of Standardization LEAP: Lifestyle Enrichment for Alzheimer Prevention
PEDro scale: physiotherapy evidence database scale
PRISMA: Preferred Reporting Items for Systematic Reviews and Meta-Analysis
SMD: standardized mean difference
