# Supplementary material for: Web-Based Multidomain Lifestyle Programs for Brain Health: Comprehensive Overview and Meta-Analysis
Source: JMIR Ment Health. 2019 Apr 9;6(4):e12104. doi: 10.2196/12104 (PMC6477576; doi:10.2196/12104)
Supplement: Multimedia Appendix 1 [file mental_v6i4e12104_app1.pdf]

Supplementary file 1: Search strategy

This Supplementary file provides the search strategy details, performed June 5th, 2018 (read from bottom-up).

Search strategy in PubMed June 5th, 2018 (read from bottom-up).

Set	Search terms	Result
#6	#5 NOT (("Adolescent"[Mesh] OR "Child"[Mesh] OR "Infant"[Mesh] OR adolescen*[tiab] OR child*[tiab] OR schoolchild*[tiab] OR infant*[tiab] OR girl*[tiab] OR boy*[tiab] OR teen[tiab] OR teens[tiab] OR teenager*[tiab] OR youth*[tiab] OR pediatr*[tiab] OR paediatr*[tiab] OR puber*[tiab]) NOT ("Adult"[Mesh] OR adult*[tiab] OR man[tiab] OR men[tiab] OR woman[tiab] OR women[tiab]))	3571
#5	#1 AND #2 AND #3 AND #4	3970
#4	"Cognition"[Mesh:NoExp] OR "Cognitive Aging"[Mesh] OR "Memory"[Mesh] OR cognitive[tiab] OR cognition[tiab] OR aging[tiab] OR ageing[tiab] OR memory[tiab] OR brain health[tiab]	704016
#3	"life style"[mesh] OR "health behavior"[mesh] OR "Stress, Psychological"[Mesh] OR "exercise"[mesh] OR life style*[tiab] OR lifestyle*[tiab] OR weight[tiab] OR behavior*[tiab] OR behaviour*[tiab] OR mood[tiab] OR nutrition[tiab] OR diet[tiab] OR exercise*[tiab] OR stress[tiab] OR sleep[tiab] OR sedentary[tiab] OR activit*[tiab] OR healthy[tiab] OR brain health[tiab] OR multimodal[tiab] OR multidomain[tiab]	5581090
#2	"Early Medical Intervention"[Mesh] OR "health education"[mesh] OR "Health Promotion"[Mesh] OR support[tiab] OR advice[tiab] OR counseling[tiab] OR counselling[tiab] OR intervention*[tiab] OR training[tiab] OR health education[tiab] OR coaching[tiab] OR program*[tiab] OR initiative*[tiab] OR prevention[tiab]	2978484
#1	"Telemedicine"[Mesh:NoExp] OR "Multimedia"[Mesh] OR "Cell phones"[Mesh] OR "Public health informatics"[Mesh] OR "Computers, handheld"[Mesh] OR "Mobile Applications"[Mesh] OR "Internet"[Mesh] OR mobile health*[tiab] OR mhealth*[tiab] OR m health*[tiab] OR telehealth*[tiab] OR tele health[tiab] OR telemedic*[tiab] OR tele medic*[tiab] OR telemonitor*[tiab] OR tele monitor*[tiab] OR ehealth*[tiab] OR e-health*[tiab] OR telecare[tiab] OR tele care[tiab] OR digital health[tiab] OR app[tiab] OR apps[tiab] OR smartphone*[tiab] OR phone application*[tiab] OR telephone application*[tiab] OR mobile application*[tiab] OR mobile technolog*[tiab] OR health technolog*[tiab] OR health application*[tiab] OR internet*[tiab] OR world wide web*[tiab] OR webportal*[tiab] OR web portal*[tiab] OR ipad[tiab] OR ipads[tiab] OR sms[tiab] OR mms[tiab] OR text messag*[tiab] OR pda[tiab] OR laptop*[tiab] OR palmtop*[tiab] OR palm top*[tiab] OR personal digital assistant*[tiab] OR interactive voice response*[tiab] OR multimedia[tiab] OR Mhapps[tiab] OR iphone*[tiab] OR android[tiab] OR game*[tiab] OR gamification[tiab] OR whatsapp*[tiab] OR e-coach*[tiab] OR wearable*[tiab] OR social media[tiab] OR online social network*[tiab]	231055

Search strategy in Embase.com June 5th, 2018 (read from bottom-up).

Set	Search terms	Result
#7	#6 NOT ('conference abstract'/it OR 'conference review'/it OR 'letter'/it)	3719
#6	#5 NOT (('adolescent'/exp OR 'child'/exp OR adolescent*:ab,ti,kw OR child*:ab,ti,kw OR schoolchild*:ab,ti,kw OR infant*:ab,ti,kw OR girl*:ab,ti,kw OR boy*:ab,ti,kw OR teen:ab,ti,kw OR teens:ab,ti,kw OR teenager*:ab,ti,kw OR youth*:ab,ti,kw OR pediatr*:ab,ti,kw OR paediatr*:ab,ti,kw OR puber*:ab,ti,kw) NOT ('adult'/exp OR	5106

	'aged'/exp OR 'middle aged'/exp OR adult*:ab,ti,kw OR man:ab,ti,kw OR men:ab,ti,kw OR woman:ab,ti,kw OR women:ab,ti,kw))	
#5	#1 AND #2 AND #3 AND #4	5734
#4	'cognition'/de OR 'memory'/exp OR 'cognitive aging'/exp OR cognitive:ab,ti,kw OR cognition:ab,ti,kw OR aging:ab,ti,kw OR ageing:ab,ti,kw OR memory:ab,ti,kw OR ‘brain health’:ab,ti,kw	969410
#3	'lifestyle'/exp OR 'health behavior'/exp OR 'stress'/exp OR 'exercise'/exp OR ‘life style*’:ab,ti,kw OR lifestyle*:ab,ti,kw OR behavior*:ab,ti,kw OR behaviour*:ab,ti,kw OR mood:ab,ti,kw OR nutrition:ab,ti,kw OR diet:ab,ti,kw OR exercise*:ab,ti,kw OR stress:ab,ti,kw OR sleep:ab,ti,kw OR sedentary:ab,ti,kw OR activit*:ab,ti,kw OR healthy:ab,ti,kw OR multimodal:ab,ti,kw OR multidomain:ab,ti,kw	6915434
#2	'early intervention'/exp OR 'health education'/exp OR 'health promotion'/exp OR support:ab,ti,kw OR advice:ab,ti,kw OR counseling:ab,ti,kw OR counselling:ab,ti,kw OR intervention*:ab,ti,kw OR training:ab,ti,kw OR ‘health education’:ab,ti,kw OR coaching:ab,ti,kw OR program*:ab,ti,kw OR initiative*:ab,ti,kw OR prevention:ab,ti,kw	3874022
#1	'telehealth'/de OR 'telemedicine'/de OR 'telemonitoring'/exp OR 'internet'/exp OR 'multimedia'/exp OR 'mobile phone'/exp OR 'microcomputer'/exp OR 'mobile application'/exp OR 'e health':ab,ti,kw OR ehealth:ab,ti,kw OR ‘mobile health*’:ab,ti,kw OR mhealth*:ab,ti,kw OR ‘m health*’:ab,ti,kw OR telehealth*:ab,ti,kw OR ‘tele health’:ab,ti,kw OR telemedic*:ab,ti,kw OR ‘tele medic*’:ab,ti,kw OR telemonitor*:ab,ti,kw OR ‘tele monitor*’:ab,ti,kw OR telecare:ab,ti,kw OR ‘tele care’:ab,ti,kw OR ‘digital health’:ab,ti,kw OR app:ab,ti,kw OR apps:ab,ti,kw OR smartphone*:ab,ti,kw OR ‘phone application*’:ab,ti,kw OR ‘telephone application*’:ab,ti,kw OR ‘mobile application*’:ab,ti,kw OR ‘mobile technolog*’:ab,ti,kw OR ‘health technolog*’:ab,ti,kw OR ‘health application*’:ab,ti,kw OR internet*:ab,ti,kw OR ’world wide web*’:ab,ti,kw OR webportal*:ab,ti,kw OR ‘web portal*’:ab,ti,kw OR ipad:ab,ti,kw OR ipads:ab,ti,kw OR sms:ab,ti,kw OR mms:ab,ti,kw OR ‘text messag*’:ab,ti,kw OR pda:ab,ti,kw OR laptop*:ab,ti,kw OR palmtop*:ab,ti,kw OR ‘palm top*’:ab,ti,kw OR ‘personal digital assistant*’:ab,ti,kw OR ‘interactive voice response*’:ab,ti,kw OR multimedia:ab,ti,kw OR Mhapps:ab,ti,kw OR iphone*:ab,ti,kw OR android:ab,ti,kw OR game*:ab,ti,kw OR gamification:ab,ti,kw OR whatsapp*:ab,ti,kw OR ‘e-coach*’:ab,ti,kw OR wearable*:ab,ti,kw OR ‘social media’:ab,ti,kw OR ‘online social network*’:ab,ti,kw	311451

Search strategy in PsycINFO June 5th, 2018 (read from bottom-up).

Set	Search terms	Result
S6	S5 NOT ((ZG ("adolescence (13-17 yrs)" OR "childhood (birth-12 yrs)" OR "infancy (2-23 mo)” OR "neonatal (birth-1 mo)" OR "preschool age (2-5 yrs)" OR "school age (6-12 yrs)") OR TI (adolescen* OR child* OR schoolchild* OR infant* OR girl* OR boy* OR teen OR teens OR teenager* OR youth* OR pediatr* OR paediatr* OR puber*) OR AB (adolescen* OR child* OR schoolchild* OR infant* OR girl* OR boy* OR teen OR teens OR teenager* OR youth* OR pediatr* OR paediatr* OR puber*)) NOT (ZG ("adulthood (18 yrs & older)" OR "aged (65 yrs & older)" OR "middle age (40-64 yrs)" OR "thirties (30-39 yrs)" OR "very old (85 yrs & older)") OR TI (adult* OR man OR men OR woman OR women) OR AB (adult* OR man OR men OR woman OR women)))	3295
S5	S1 AND S2 AND S3 AND S4	3775
S4	DE ("Cognition" OR "Memory") OR TI (cognitive OR cognition OR aging OR ageing OR memory) OR AB (cognitive OR cognition OR aging OR ageing OR memory)	597169

S3	DE ("Lifestyle Changes" OR "Lifestyle" OR "Health Behavior" OR "Exercise" OR "Occupational Stress" OR "Psychological Stress" OR "Social Stress") OR TI ("life style*" OR lifestyle* OR weight OR behavior* OR behaviour* OR mood OR nutrition OR diet OR exercise* OR stress OR sleep OR sedentary OR activit* OR healthy OR "brain health" OR multimodal OR multidomain) OR AB ("life style*" OR lifestyle* OR weight OR behavior* OR behaviour* OR mood OR nutrition OR diet OR exercise* OR stress OR sleep OR sedentary OR activit* OR healthy OR "brain health" OR multimodal OR multidomain)	1501694
S2	DE ("Intervention" OR "Early Intervention" OR "Workplace Intervention" OR "Prevention" OR "Preventive Medicine" OR "Health Education" OR "Health Promotion") OR TI (support OR advice OR counseling OR counselling OR intervention* OR training OR “health education” OR coaching OR program* OR initiative* OR prevention) OR AB (support OR advice OR counseling OR counselling OR intervention* OR training OR “health education” OR coaching OR program* OR initiative* OR prevention)	1238253
S1	DE ("Telemedicine" OR "Internet" OR "Cellular Phones" OR "Computer Applications" OR "Multimedia" OR "Microcomputers" OR "Mobile Devices" OR "Social Media" OR "Online Social Networks" OR "Text Messaging") OR TI(“e health” OR ehealth OR “mobile health*” OR mhealth* OR “m health*” OR telehealth* OR “tele health” OR telemedic* OR “tele medic*” OR telemonitor* OR “tele monitor*” OR telecare OR “tele care” OR “digital health” OR app OR apps OR smartphone* OR “phone application*” OR “telephone application*” OR “mobile application*” OR “mobile technolog*” OR “health technolog*” OR “health application*” OR internet* OR “world wide web*” OR webportal* OR “web portal*” OR ipad OR ipads OR sms OR mms OR “text messag*” OR pda OR laptop* OR palmtop* OR “palm top*” OR “personal digital assistant*” OR “patient monitoring” OR “interactive voice response*” OR multimedia OR Mhapps OR iphone* OR android OR game* OR gamification OR whatsapp* OR “e-coach*” OR wearable* OR “social media” OR “online social network*”) OR AB (“e health” OR ehealth OR “mobile health*” OR mhealth* OR “m health*” OR telehealth* OR “tele health” OR telemedic* OR “tele medic*” OR telemonitor* OR “tele monitor*” OR telecare OR “tele care” OR “digital health” OR app OR apps OR smartphone* OR “phone application*” OR “telephone application*” OR “mobile application*” OR “mobile technolog*” OR “health technolog*” OR “health application*” OR internet* OR “world wide web*” OR webportal* OR “web portal*” OR ipad OR ipads OR sms OR mms OR “text messag*” OR pda OR laptop* OR palmtop* OR “palm top*” OR “personal digital assistant*” OR “interactive voice response*” OR multimedia OR Mhapps OR iphone* OR android OR game* OR gamification OR whatsapp* OR “e-coach*” OR wearable* OR “social media” OR “online social network*”)	129173
