# Supplementary figures and images for: Web-Based Multidomain Lifestyle Programs for Brain Health: Comprehensive Overview and Meta-Analysis
Source: JMIR Ment Health. 2019 Apr 9;6(4):e12104. doi: 10.2196/12104 (PMC6477576; doi:10.2196/12104)

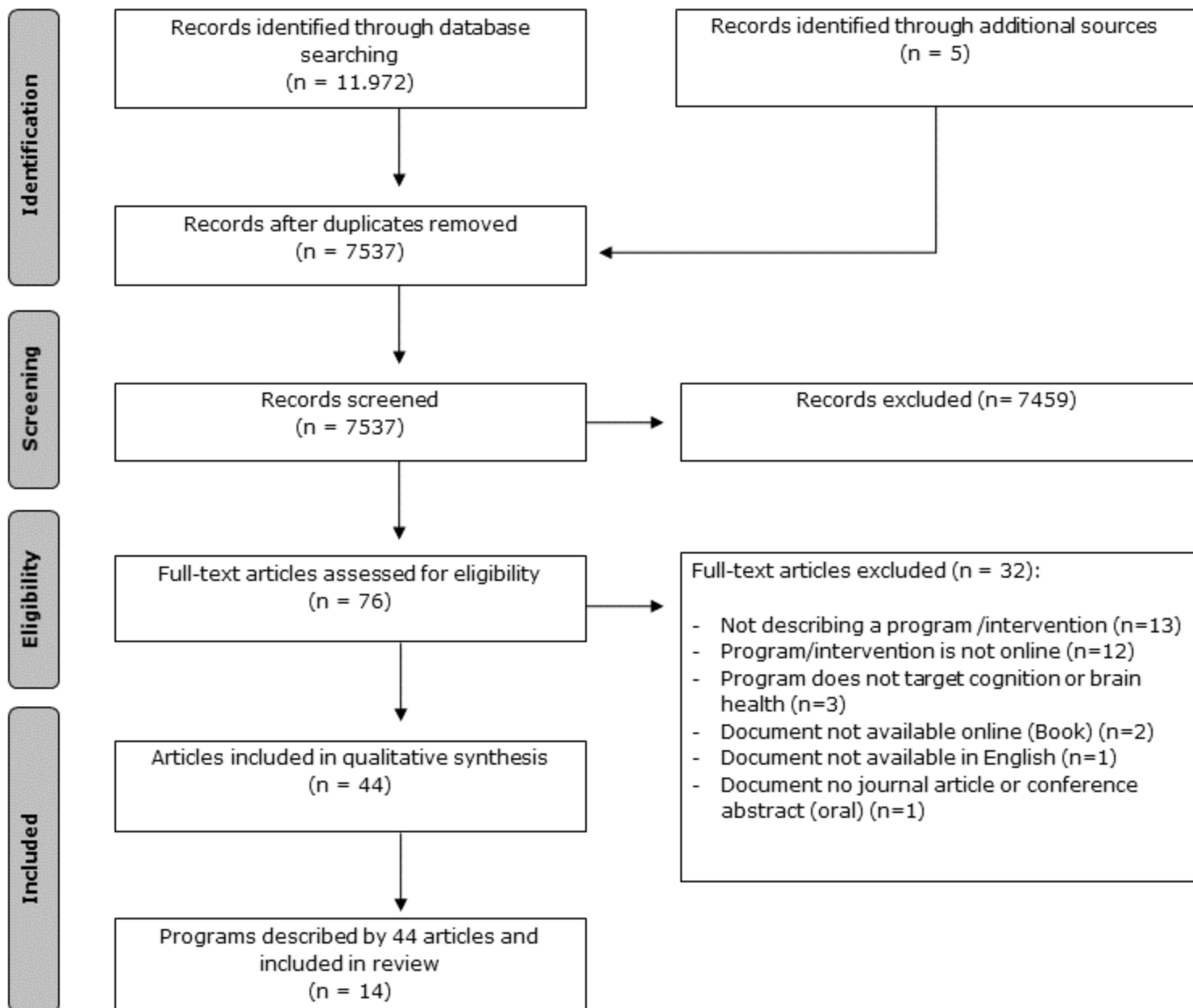

Supplement: Multimedia Appendix 2 [file mental_v6i4e12104_app2.pdf]
